# Trend of adherence to iron supplementation during pregnancy among Ethiopian women based on Ethiopian demographic and health surveys: A Multivariable decomposition analysis

**DOI:** 10.3389/fnut.2022.955819

**Published:** 2022-12-16

**Authors:** Amare Mebrat Delie, Lemma Derseh Gezie, Asaye Alamneh Gebeyehu, Gebrekidan Ewnetu Tarekegn, Achenef Asmamaw Muche

**Affiliations:** ^1^Department of Public Health, College of Health Science, Woldia University, Woldia, Ethiopia; ^2^Department of Epidemiology and Biostatistics, Institute of Public Health, University of Gondar, Gondar, Ethiopia; ^3^Department of Public Health, College of Health Science, Debre Tabor University, Debre Tabor, Ethiopia

**Keywords:** adherence, EDHS, pregnant women, iron supplement, decomposition analysis

## Abstract

**Background:**

Iron deficiency is one of the significant factors of anemia during pregnancy. Iron supplementation is the main method of prevention and control of iron deficiency anemia, and its effectiveness depends on adherence to the iron supplementation.

**Methods:**

This study was based on a secondary analysis of 2005, 2011, and 2016 EDHS data. After the data was weighted using sampling weight, 696, 1,282, and 3,096 in 2005, 2011, and 2016 EDHS data, respectively, were used for the final analysis. The data were edited, cleaned, coded, managed, and analyzed using StataCorp version 16 software. A logit-based multivariable decomposition analysis was used to identify variables significantly associated with the change in the adherence level during pregnancy.

**Results:**

Adherence levels increased from 1.1% (95% CI; 0.4, 2.7) in 2005 EDHS to 12.4% (95% CI; 10.9, 14.1) in 2016 EDHS. About 30.9% of the overall change in the adherence level to iron supplement use during pregnancy was due to the difference in women's sociodemographic-related variables. After adjusting for these compositional changes, ~69.1% of the change in the adherence level was because of the difference in the coefficients (behavior-related variables). Among the behavioral characteristics, women's age-group, rich wealth index, and secondary and above-secondary educational status of their husbands had a statistically significant effect on the positive change in the adherence level of pregnant mothers.

**Conclusion:**

The adherence level to iron supplement use during pregnancy has increased significantly over the last decade in Ethiopia. Both the compositional and behavioral characteristics of women play a major role in the increasing trend of adherence levels.

## Background

The World Health Organization (WHO) recommends that pregnant women take daily oral iron–folic acid supplementation with 30–60 mg of iron and 0.4 mg of folic acid to prevent maternal anemia, puerperal sepsis, low birth weight, and preterm birth and to increase the demand for iron–folic acid as well as other nutrients during pregnancy to meet the daily requirements for the fetus's life development and growth (1).

Anemia caused by a lack of iron–folic acid is a worldwide public health problem, particularly in low- and middle-income countries (LMICs). During pregnancy and post-pregnancy, iron–folic acid is a vital micronutrient for physiological function, growth, and development, as well as for maintaining life for the mother and the fetus (2). Increased iron–folic acid supplementation during pregnancy helps decrease the risk of anemia, hemorrhage, sepsis, maternal mortality, and productivity (3). However, if provided an excess of iron folic acid may cause peroxidative damage through production of reactive oxygen species including loss of functional integrity, and decreased turnover of epithelial cells, as also with marked mucosal cell death (4–6).

In sub-Saharan Africa, the overall prevalence of at least 90 days of iron supplementation during pregnancy was 28.7%, ranging from 1.4% in Burundi to 73.0% in Senegal (7). According to the Ethiopian Demographic and Health Survey (EDHS) of 2005, 2011, and 2016, <0.1, 1.1, and 5.1% of pregnant women, respectively, used IFA supplements for the recommended period during their pregnancy (pregnant women should take approximately 30–60-mg IFA during their pregnancy) (8–10). Different studies carried out in Ethiopia based on self-reported adherences showed that ~38.3–47.6% of pregnant women were adherent to recommended IFA supplementation (11–13).

By the 2015 EC, the Ethiopian National Nutrition Strategy (NNS) aims to increase the proportion of mothers receiving iron supplements for more than 90 days throughout pregnancy and postpartum to 50% (14). Despite this intervention strategy, according to the Mini EDHS 2019, the prevalence of iron supplement adherence was only 11%, which was far too low (15).

Miscarriage, stillbirths, preterm, low birth weight, congenital deformities, and perinatal mortality are some risks of iron–folic acid deficiency during pregnancy (16, 17). According to a systematic review, iron–folic acid supplementation could minimize 50% of iron deficiency anemia in pregnant women and reduce the risk of low birth weight by 19% (18).

Iron deficiency anemia is the most common hematologic disorder in pregnant women and children worldwide, particularly in LMICs. Low intake of iron–folic acid supplement during pregnancy has been linked to a higher risk of adverse birth outcomes such as neural tube defects, cardiac defects, and endocrine disorders. Iron–folic acid supplementation is the currently recommended strategy to prevent the adverse outcomes (19, 20). However, blind iron supplementation should be avoided, and iron supplementation provided on a need basis. Approximately 38.2% of pregnant women were anemic globally, among which Africa accounts for the highest magnitude-−44.6% for 44.6% (21). Furthermore, anemia among pregnant women is a serious public health problem in Ethiopia, with an overall prevalence of 62.7%, and this high prevalence has been linked to nutrition deficiencies (iron, folate, and vitamin B12) and non-nutritional factors in developing countries (parasitic infections and genetic diseases). Among the aforementioned causes, iron deficiency has been assumed to account for roughly half of anemia cases, and mothers with inadequate diets and who did not receive the recommended days of prenatal supplements were more susceptible to anemia (22, 23).

ANC utilization, educational status, wealth index, anemia complications, knowledge about IFA, family size, history of abortion, and receiving health education are factors that were significant predictors of decreased iron supplement adherence in various studies (7, 12, 24, 25). A previous study used only one-point survey data, making it difficult to observe trends and identify potential predictors that have contributed to influencing iron supplement adherence over time. Studying the change in iron supplement adherence using multilevel decomposition analysis to identify determinant predictors associated with the change in iron supplement adherence over time has become relevant to target interventions to work on predictors contributing to increased iron–folic acid adherence and could critically inform policies and programs aimed at improving the health of pregnant women and children in Ethiopia.

## Materials and methods

### Source of data

The study was a secondary analysis of the Ethiopian Demographic and Health Survey (EDHS) of 2005, 2011, and 2016. The DHS used a two-stage stratified sampling technique, selecting individuals in two stages based on the 1994 Population and Housing Census (PHS) frame for EDHS 2005 and the 2007 PHS frame for EDHS 2011 and 2016. Each region was stratified by separating it into urban and rural areas. Ethiopia is composed of nine national regions and two administrative towns, so the total number of strata created was 21 because Addis Ababa is entirely urban. A total of 540 enumeration areas (EAs) (395 in rural areas) for EDHS 2005, 624 EAs (534 in rural areas) for EDHS 2011, and 645 EAs (445 in rural areas) for EDHS 2016 were selected in the first stage, with proportional to EA size and independent selection in each stratum. In the second stage, households were selected using a systematic approach per enumeration area. The full EDHS report included details on the sampling design for the EDHS (26). In 2005, 2011, and 2016 EDHS, the sample was drawn from all pregnant women who had at least one live birth in the previous 5 years.

### Study variables

#### Dependent variables

In this study, adherence to iron supplementation was taken as the dependent variable. It was defined as an intake of iron tablets or syrup supplements daily for 90 days during a recent pregnancy (27–29). The ith women in the dependent variable are represented by a random variable Yi, with two possible values coded 1 and 0. As a result, the ith women Y_i_ response variable was measured as a dichotomous variable, with possible values of Y_i_ = 1 if ith women had taken iron tablets or syrup supplements for 90 days, and Y = 0 if women had not taken 90 days.

#### Independent variables

The sociodemographic and socioeconomic variables such as age of the women, residence, parity, employment status, educational status of their husbands, educational status of women, and wealth index were used to explain the adherence level. Among health service-related factors, number of ANC visits for the indexed pregnancy, mass media exposure (indexed from television, newspaper, and radio), complications at the time of last ANC visit, timing of first ANC visit, pregnancy status (wanted or not wanted), and accessibility of healthcare were used as independent variables in this study.

The wealth index was categorized into three categories by combining the poorest and poorer into the first category “poor;” middle wealth level into the second category “middle;” and richer and richest into the third category called “rich,” based on the previous literature (30, 31).

### Data collection procedure

Since the study was based on the EDHS, data were obtained from the official database of the DHS program at www.measuredhs.com. After requesting permission online and explaining the purpose of the study, permission was granted. The raw data were collected from all over the country on pregnant mothers and mothers who had at least one live birth in the previous 5 years about their iron–folic acid adherence level during pregnancy.

### Data quality assurance

Data from the EDHS 2005, 2011, and 2016 were accessed from the DHS official database and were appended together after extracting the relevant variables for trend and decomposition analysis. STATA version 14 software was used to clean, compute, recode, and handle the missed data. Before any statistical analysis, the data were weighted using sampling weight, primary sampling units, and strata to restore the representativeness of the data and to instruct STATA to take the sampling design into account when calculating the standard sampling errors to make the statistical estimates reliable. To describe the study population, summary statistics were computed.

### Trend and multivariable decomposition analysis

The adherence level trend was examined for the EDHS 2005–2011, 2011–2016, and 2005–2016 periods separately. A multivariable decomposition analysis of the change in the adherence level to iron supplement use was used to answer the question of how the adherence level to iron supplement use responds to changes in women's characteristics and how these factors shape the differences in adherence to iron supplement use across three consecutive EDHSs conducted at different time points. To identify factors associated with the change in the adherence level over the last 10 years, a logit-based multivariable decomposition analysis was used. The change in the adherence level over time can be attributed to the survey composition and differences in the effect of the selected predictors. As a result, the observed difference in the adherence level between surveys can be decomposed additively into a characteristic (endowment) component and a coefficient (or effect) component. For logistic regression, the logit or log-odds of iron supplement adherence is expressed as $(\bar{Y_{A}}-\bar{Y_{B}}=F\left(X_{A}\beta_{A}\right)-F(X_{B}=\underset{E}{\underset{︸}{\left\{\bar{F(\chi_{A}\beta_{A})}-\bar{F(\chi_{B}\beta_{A})}\right\}}}+\underset{C}{\underset{︸}{\left\{\bar{F(\chi_{B}\beta_{A})}-\bar{F(\chi_{B}\beta_{B})}\right\}}}$


$$\begin{array}{l} =logit\left(\text{Δ}Y^{2016-2005}\right)=\left(X^{2016}-X^{2005}\right)\beta^{2016}\\ \;+\left(\beta^{2016}-\beta^{2005}\right)X^{2005}. \end{array}$$

The component labeled E denotes the portion of the differential attributable to differences in endowments or characteristics, which is commonly referred to as the explained component or characteristic effects. The C component is the portion of the differential attributable to differences in coefficients or effects, which is also known as the unexplained component or coefficient effects. Group A was designated as the comparison group, while group B was designated as the reference group. “E” represents the difference in outcomes from A's point of view (i.e., the expected difference of group A gives group B's covariate distribution).

The equation can be written as follows:


$$\begin{array}{l} logit\left(\text{Δ}Y^{2016-2005}\right)=E+C=\overset{k}{\underset{k=1}{\sum }}E_{k}+\overset{k}{\underset{k=1}{\sum }}C_{k}\\ logit\left(\text{Δ}Y^{2016-2005}\right)=\left(X^{2016}-X^{2005}\right)\beta^{2016}\\ \;+\left(\beta^{2016}-\beta^{2005}\right)X^{2005}\\ \;=\left[\beta_{0A}-\beta_{0B}\right]{+}_{ijB}^{*}\left[\beta_{ijA}-\beta_{ijB}\right]\\ \;+\sum \beta_{ijB}^{*}\left[X_{ijA}-X_{ijB}\right], \end{array}$$

where *X*_*ijB*_ is the portion of the j^*th*^ category of the i^*th*^ covariate in the EDHS 2005, *X*_*ijA*_ is the proportion of the j^*th*^ category of the i^*th*^ determinant in the EDHS 2016, *β*_*ijB*_ is the coefficient of the j^*th*^ category of the i^*th*^ determinant in the EDHS 2005, *β*_*ijA*_ is the coefficient of the j^*t*^*h* category of the i^*th*^ determinant in the EDHS 2016, *β*0*B* is the intercept in the regression equation fitted to the EDHS 2005, and *β*_0*A*_ represents the intercept in the regression equation fitted to the EDHS 2016.

The recently developed multivariate decomposition for the non-linear model was used for the decomposition analysis of iron supplementation (32).

## Results

### Respondents' socioeconomic and demographic characteristics

According to the findings of this study, the majority of respondents (79.1% in the EDHS 2005, 72.1% in the EDHS 2011, and 80.8% in the EDHS 2016) lived in rural areas of the country. Almost half of the respondents (in all three EDHSs) were wealthy and between the ages of 25 and 34 years. Furthermore, a large proportion of respondents (in all EDHSs) had no formal education, implying that the proportion of respondent women with secondary or higher education was very low (ranging from 12.4 to 14.9%). In the EHDS 2005, 2011, and 2016, ~58.5, 62.8, and 69.1% of the respondents had given birth to one to four children, respectively. In addition, more than half of the respondents from the EDHS 2011 and 2016 were employed women. However, only 35.1% of the respondents were employed in the EDHS 2005 (Table 1).

**Table 1:** Proportion of respondents by sociodemographic and socioeconomic characteristics in Ethiopia based on the EDHS 2005–2016.

		**Proportion (%)**		
**Characteristics of respondent**	**Categories**	**2005 EDHS *n* = 696**	**2011 EDHS *n* = 1,282**	**2016 EDHS *n* = 3,096**
Residence	Rural	79.1	72.1	80.8
	Urban	20.9	27.9	19.2
Age category	15–24 yrs.	22.7	23.9	25.6
	25–34 yrs.	51.6	50.6	53.3
	35–49 yrs.	25.7	25.5	21.1
Educational status of women	No education	65.4	54.5	53.0
	Primary	20.4	33.1	32.1
	Secondary or above	14.2	12.4	14.9
Educational status of husband	No education	41.7	36.6	40.5
	Primary	37.0	45.2	38.8
	Secondary or Higher	21.3	18.2	20.7
Wealth	Poor	22.5	27.4	36.1
	Middle	22.6	17.3	19.1
	Rich	54.9	55.2	44.8
Employment status of women	Not employed	64.9	38.8	48.4
	Employed	35.1	61.2	51.6
Parity	1–4	58.5	62.8	69.1
	5–9	36.4	33.7	29.1
	10+	5.1	3.5	1.8

yrs., years 10+=10 and above children; n, weighted sample size.

### Respondents' health service-related characteristics

The pregnancy status, number of ANC visits, timing of first ANC visit, accessibility of healthcare, exposure to mass media, and who told about pregnancy complications were used to examine respondents' health service-related characteristics. In the EDHS 2016 and 2005, a majority (75.7%) and more than half (57.6%) of the women, respectively, responded that a child was desired during pregnancy. More than half of the EDHS 2011 and 2016 respondents (54 and 55%, respectively) made four or more antenatal care visits. However, the proportion of EDHS 2005 respondents who made four or more antenatal care visits was about 46% (Table 2).

**Table 2:** Proportion of respondents with health service-related characteristics in Ethiopia based on the EDHS 2005–2016.

		**Proportion (%)**		
**Characteristics of respondent**	**Categories**	**2005 EDHS *n* = 696**	**2011 EDHS *n* = 1,282**	**2016 EDHS *n* = 3,096**
Pregnancy status	Wanted	57.6	68.8	75.7
	Not wanted	42.4	31.2	24.3
Number of antenatal care visit	≥4	46.0	53.6	55.2
	<4	54.0	46.4	44.8
Timing of first antenatal care visit	<4 month	23.7	30.7	35.2
	≥4 month	76.3	69.3	64.8
Problem in Accessing healthcare	Not a big problem	73.7	82.8	80.4
	A Big problem	26.3	17.2	19.6
Exposure to mass media	Yes	20.4	32.3	24.3
	No	79.6	67.7	75.7
Told about pregnancy complication	Yes	35.8	26.5	51.1
	No	64.2	73.5	48.9

n, weighted sample size.

### Trends in the proportion of adherence levels to iron supplements

**Figure 1:**
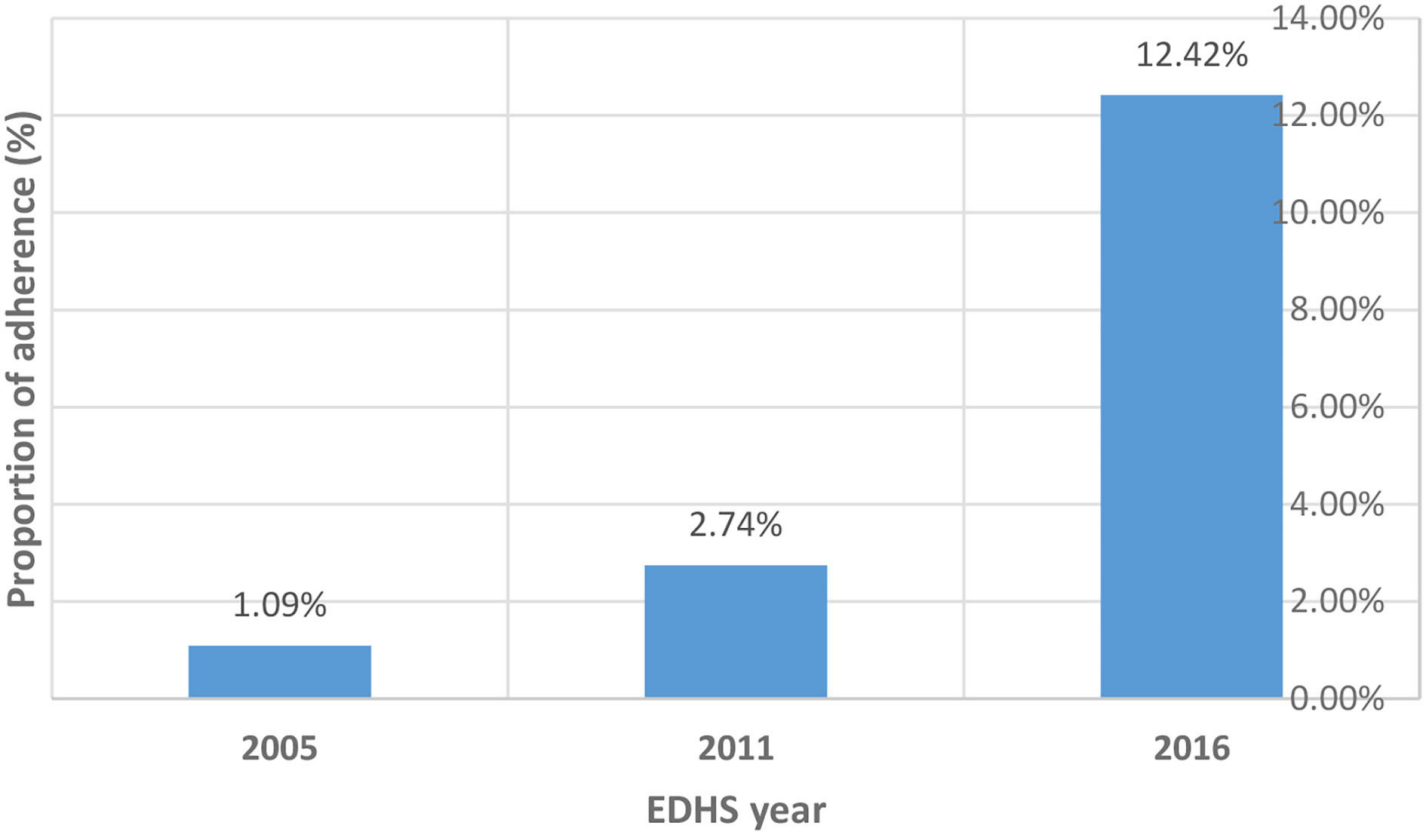
Proportion of adherence levels across three consecutive EDHS.

The proportion of respondents who took iron tablets or syrup for 90 days and above increased from 1.1% (95% CI; 0.4, 2.7) in the EDHS 2005 to 12.4% (95% CI; 10.9, 14.1) in the EDHS 2016. The trends of adherence were positively increased in both phases: phase 1 (from EDHS 2005 to 2011) and phase 2 (from EDHS 2011 to 2016). However, a larger increase in the proportion of adherence levels was observed in phase 2 (9.7%) than in phase 1 (1.6%). There was an 11.3% total increase in the proportion of adherence levels between the EDHS 2005 and 2016 (Figure 1).

#### Proportion of adherence levels based on sociodemographic and socioeconomic characteristics

The point percent differences in the proportion of adherence levels between EDHS 2011–2005, 2016–2011, and 2016–2005 for sociodemographic and socioeconomic variables with their respective categories are given in Table 3. Some categories of sociodemographic variables showed a major increment in adherence levels. For example, an increase in adherence levels was observed among urban residents during the second phase of the study period (2011–2016), with a 14.0 percentage point increase compared with a 2.7 percentage point increase during the first phase (2005–2011).

**Table 3:** Proportion of adherence levels to iron supplements based on the sociodemographic and socioeconomic characteristics of the respondents in Ethiopia (EDHS 2005–2016).

**Variables**	**Categories**	**2005** ***n* = 696**	**2011** ***n* = 1,282**	**2016** ***n* = 3,096**	**Percentage point difference in the adherence level**		
					**2011–2005 EDHS**	**2016–2011** ** EDHS**	**2016–2005** ** EDHS**
Residence	Rural	1.1	2.4	11.2	1.3	8.8	10.1
	Urban	1.0	3.7	17.7	2.7	14.0	16.7
Age category	15–24 yrs.	2.6	4.8	13.5	2.2	8.7	10.9
	25–34 yrs.	0.3	1.9	11.5	1.6	9.6	11.2
	35–49 yrs.	1.2	2.6	13.3	1.4.	10.7	12.1
Educational status women	No formal education	1.3	2.8	10.0	1.5	7.2	8.7
	Primary	0.1	2.1	14.0	2.0	11.9	13.9
	Secondary or above	1.2	3.7	18.4	2.5	14.7	17.2
Educational status husband	No formal education	1.7	2.3	9.9	0.6	7.6	8.2
	Primary	0.4	3.2	12.8	2.8	9.6	12.4
	Secondary or above	0.8	0.4	17.1	−0.4	16.7	16.3
Wealth	Poor	1.9	2.8	11.4	0.9	8.6	9.5
	Medium	1.7	2.8	8.8	1.1	6.0	7.1
	Rich	0.3	2.6	15.0	2.3	12.4	14.7
Employment status	Unemployed	0.4	2.7	11.1	2.3	8.4	10.7
	Employed	2.7	1.6	13.7	−1.1	12.1	11.0
Parity	1–4	1.3	3.0	13.0	1.7	10.0	11.7
	5–9	0.6	2.4	11.5	1.8	9.1	10.9
	10+	2.1	0.5	4.4	−1.6	3.9	2.3

N, weighted sample size; yrs., years 10+ = 10 and more children born.

Respondents with secondary or higher education had a 17.2 percentage point increase in adherence levels between the EDHS 2005 and 2016. In all age-groups, there was an increase in adherence levels to iron supplements. Women aged 35–49 years showed a larger increase in adherence levels (12.1%) than women aged 15–24 years (10.9%) and 25–34 years (11.2%). Regarding household wealth status, the adherence level of women from rich households increased by 14.7 percentage points between the EDHS 2005 and 2016, which is higher than that for women from poor households (9.5 percentage points). Moreover, the adherence level of women who had given birth to one to four children increased by 11.7% compared with 2.3% of women who had given birth to 10 or more children among the EDHS 2005 and 2016. Lastly, the adherence level of employed women increased by 11% between the EDHS 2005 and 2016 (Table 3).

#### Proportion of adherence level based on health service-related characteristics of respondents

The point percent differences in the proportion of adherence levels from the EDHSs 2011 to 2005, 2016 to 2011, and 2016 to 2005 for health-related variables with their respective categories are given in Table 4. In all categories of health-related variables, higher point differences were observed in phase 2, while lower point differences were observed in phase 1 (Table 4). Women who made four or more ANC visits increased their adherence levels by 15.1%, nearly two times the women who made less than four antenatal care visits (6.7%). Likewise, the proportion of adherence levels of women who made the first antenatal care visit within 4 months increased by 15.8%, which is also two times the increment of women who made the first ANC visit after 4 months (8.6%).

**Table 4:** Proportion of respondents in Ethiopia who adhere to iron supplementation based on health-related characteristics (EDHS 2005–2016).

**Variables**	**Categories**	**2005 EDHS *n* = 696**	**2011 EDHS *n* = 1,282**	**2016 EDHS *n* = 3,096**	**Percentage point difference in adherence levels**		
					**2011–2005 EDHS**	**2016–2011** ** EDHS**	**2016–2005** ** EDHS**
Pregnancy status	Wanted	0.4	1.5	12.8	1.1	11.3	12.4
	Not wanted	1.5	3.3	11.2	1.8	7.9	9.7
Number of ANC visit	≥4 ANC	2.6	3.0	17.7	0.4	14.7	15.1
	<4 ANC	0.4	2.5	7.1	2.1	4.6	6.7
Timing of first ANC visit	<4 months	1.5	2.7	17.3	1.2	14.6	15.8
	≥4 months	2.4	2.5	11.0	0.1	8.5	8.6
Problem in accessing healthcare	Not big problem	1.1	3.0	13.5	1.9	10.5	12.4
	Big problem	1.1	1.3	7.4	0.2	6.1	6.3
Exposure o mass media	Yes	0.6	3.8	18.2	3.2	14.4	17.6
	No	1.2	2.2	10.5	1.0	8.3	9.3
Told about pregnancy complication	Yes	2.4	3.0	16.7	0.6	13.7	14.3
	No	1.3	2.4	9.8	1.1	7.4	8.5

ANC, antenatal care visit; n, sample size.

Among respondent women who reported that there were no serious problems in accessing healthcare, the adherence level increased by 12.4%, which is higher than that of women who reported that accessing healthcare was a serious problem (6.3%). Women who were exposed to mass media, on the other hand, increased their adherence levels by 17.6 % compared with women who were not exposed to mass media (9.3 %) among the EDHS 2005 and 2016. From the EDHSs 2005 to 2016, the level of adherence in women who received information about pregnancy complications increased by 14.3% (Table 4).

### Decomposition analysis

To estimate the explained and unexplained sources of variation, a decomposition analysis was employed, and the results showed that both the explained and unexplained sources of variation significantly contributed to the change in the level of adherence to iron supplement use (Tables 5, 6) between EDHSs 2011–2016 and 2005–2016. Nevertheless, the compositional and behavioral effects did not significantly contribute to the change in the adherence level of iron supplement use during pregnancy between EDHS 2005 and 2011.

**Table 5:** Decomposition analysis of changes in adherence levels to iron supplements during pregnancy based on the EDHS 2005–2016.

**Adherence**		**Due to difference in characteristics**		**Due to difference in coefficients**	
**Characteristics**	**Categories**	**Coefficient (CI)**	**Percent**	**Coefficient (CI)**	**Percent**
Residence	Urban	0	0	0	0
	Rural	0.00003 (−0.00050, 0.00057)	0.0	−0.00406 (−0.01416, 0.00604)	−3.5
Age category	15–24	0	0	0	0
	25–34	−0.00185 (−0.00433, 0.00064)	−1.6	0.03267 (−0.01416, 0.00604)	28.3
	35–49	0.00020 (−0.00255, 0.00295)	0.2	0.010157 (0.00239, 0.01793)	8.8
Educational level of husband	No education	0	0	0	0
	Primary education	0.00083 (−0.00127, 0.00293)	0.7	0.00357 (−0.01493, 0.02208)	3.1
	Secondary and above education	0.00007 (−0.00022 0.00036)	0.1	0.00522 (0.00028, 0.01016)	4.5
Educational status of women	No education	0	0	0	0
	Primary	0.00603 (−0.00118, 0.01323)	5.2	0.00211 (−0.00429, 0.00850)	1.8
	Secondary and above	0.00059 (−0.00065, 0.00182)	0.5	−0.00436 (−0.00923, 0.00051)	−3.8
Wealth index	Poor	0	0	0	0
	Medium	0.00140 (−0.00039, 0.00319)	1.2	−0.00477 (−0.0096, 0.00008)	−4.1
	Rich	0.00256 (−0.00039, 0.00319)	2.2	0.03077 (0.00225, 0.05929)	26.6
Employment status	Not Employed	0	0	0	0
	Employed	0.00646 (−0.00153, 0.01445)	5.6	−0.01587 (0.02304, −0.00870)	−13.7
Parity	1–4	0	0	0	0
	5–9	−0.00065 (−0.00431, 0.00301)	−0.6	−0.00847 (−0.02172, 0.00479)	−7.3
	10+	0.00463 (−0.00070, 0.00996)	4.0	−0.00331 (−0.00529, −0.00133)	−2.9

CI = 95% confidence interval for coefficients.

**Table 6:** Decomposition analysis of change in the adherence level to iron tablets or syrup use during pregnancy based on health service–related factors in Ethiopia (EDHS 2005–2016).

**Adherence characteristics**		**Difference due to characteristics**		**Difference due to coefficient**	
		**Coefficient (CI)**	**Percent**	**Coefficient (CI)**	**Percent**
Exposure to mass media	No	0	0	0	0
	Yes	0.00073 (−0.00088, 0.00233)	0.6	0.00177 (−0.00823, 0.01176)	1.5
Number of ANC	0–3	0		0	0
	≥4	0.01269 (0.00524, 0.02014)	11.0	−0.01214 (−0.02075, −0.00353)	−10.5
Timing of first ANC	<4 month	0	0	0	0
	≥4 month	0.00586 (−0.00067, 0.01240)	5.1	0.00009 (−0.00610, 0.00629)	0.1
Problem in Accessing healthcare	A problem	0	0	0	0
	Not a problem	0.00226 (−0.00055, 0.00506)	1.9	0.02269 (−0.00265, 0.04803)	19.6
Pregnancy status (when became pregnant)	Not wanted	0	0	0	0
	Wanted	0.00375 (−0.00536, 0.01286)	3.2	0.00451 (−0.00916, 0.01817)	3.9
Told about pregnancy complications	No	0	0	0	0
	Yes	0.01158 (0.00274, 0.02042)	10.0	−0.00078 (−0.00930, 0.00774)	−0.7
Total		0.03569 (0.02373, 0.04765)	30.9	0.07988 (0.06025, 0.09951)	69.1

CI = 95% confidence interval for coefficients.

#### Due to compositional effects and behavioral effects

According to the results of the decomposition analysis, approximately one-third (30.9 %) of the overall change in the adherence level to iron supplement use during pregnancy between EDHSs 2005 and 2016 was due to differences in characteristics (compositional factors). Women who made four or more ANC visits and were informed about pregnancy complications contributed 11 and 10%, respectively, to the positive change in the adherence level between the EDHSs 2005 and 2016. In addition to compositional effects, the difference in coefficients accounted for nearly two-thirds (69.1 %) of the change in the adherence level (behavioral characteristics). Women in age categories of 25–34 and 35–49 years, those from affluent households, and those with a husband with a secondary or higher educational status had a significant positive contribution to the change in adherence behavior between the EDHSs 2005 and 2016. About 26.6% of women in the age category of 25–34 years, 8.3% in the age category of 35–49 years, 26.6% from rich households, and 4.5% with husbands with secondary/educational status had contributed to a positive change in adherence behavior of respondents between EDHSs 2005 and 2016. Women who desired a child had a 3.2% positive contribution for an increase in adherence behavior between EDHSs 2011 and 2016 (Tables 5, 6).

## Discussion

During phase 1 (EDHS 2005–2011), the proportion of adherence increased from 1.09% (95% CI; 0.44, 2.67) to 2.71 % (95% CI; 1.79, 4.07). It also increased from 2.71 % (95% CI; 1.79, 4.07) in the EDHS 2011 to 12.42 % (95% CI; 10.90, 14.12) in the EDHS 2016. This could be due to the Ethiopian NNS key goal of increasing the proportion of pregnant women who receive iron–folate supplements for more than 90 days during pregnancy and the postpartum period to 50% by 2023 (14).

The decomposition analysis revealed that compositional effects accounted for approximately 30.9% of change in adherence levels (between the EDHSs 2005 and 2016). Among the compositional effects, women who had four or more ANC visits had an 11% significant positive contribution to the overall change in the adherence level compared with women who had less than four ANC visits. Furthermore, women who reported pregnancy complications had a 10% positive contribution to an increase in adherence levels over the last decade. It is obvious that an adequate number of ANC visits and discussions about pregnancy complications are critical for increasing women's awareness of the importance of taking iron tablets or syrup for 90 days during pregnancy. As a result, over time, women who made an adequate number of ANC visits had higher levels of awareness about the minimum recommended days of taking iron tablets or syrup during pregnancy than women who did not make an adequate number of ANC visits. Furthermore, women who received information about pregnancy complications during an ANC visit were more aware of the causes, consequences, and prevention of iron deficiency anemia during pregnancy than women who did not receive such services. Previous research in Ethiopia corroborates this finding (29, 33).

The difference in coefficients accounted for the remaining 69.1% of the overall change in adherence levels. Older aged women from wealthy households and women with husbands with secondary or higher education all contributed positively to the overall change in respondents' adherence behavior. Older aged women may have more knowledge about anemia during pregnancy and have made a higher number of ANC visits, allowing them to adhere to iron supplement use during pregnancy. This could explain why women aged 25–34 and 35–49 years made a significant positive contribution to the overall change in adherence to iron supplement use when compared with women aged 15–24 years. The finding was also supported by previous studies (7, 34); women in the age-group of 35–49 years were 7% more likely to adhere to iron supplements than younger women aged 15–24 years.

Furthermore, wealthy women significantly contributed 26.6% to the increase in adherence behavior than women from poor households over the last 10 years. This could be because wealthy women have more financial resources to purchase iron tablets or syrup during pregnancy. This finding was similar to that of other studies (7, 29), which reported that wealthy women were more likely to be adherent than women from poor households.

Husbands with secondary or higher education made a 4.5% significant positive contribution to the increase in behavioral effects of adherence. The finding is supported by previous studies carried out in Ethiopia (28, 29, 35). This could be because husbands with secondary or higher education have better communicate with community leaders, health extension workers, and medical specialists than those without formal education. This communication and reading ability may have increased husbands' awareness of the benefits of taking iron supplements for recommended days during pregnancy, thereby improving their wives' adherence. This is because Ethiopian wives are mostly led by their husbands.

According to our findings, women who had 10 or more children had a 2.9% negative contribution to the change in behavior of the adherence level compared with women who had one to four children. This could be because, in general, urban communities provide better care and protection to pregnant women who have not given birth to a child. Following the birth of the first child without complications, subsequent pregnancies are regarded as low risk. Furthermore, a higher number of children ever born indicates a higher number of children in the household; as a result, women may have more responsibility and less time to visit health facilities for ANC. Despite the lack of a direct study supporting this finding, a related study (7) found that women who had ever given birth to 10 or more children were less likely to be adherent than women who had given birth to one to four children.

Between the EDHSs 2011 and 2016, women who desired pregnancy had a 19.9% positive contribution to an increase in the adherence behavior. This implies that the change in the adherence behavior among women who desired a child at the time of pregnancy was much higher than the change in the adherence behavior among women who did not desire a child during pregnancy. This could be because women who desired a child at the time of pregnancy pay more attention to their child's health by taking the minimum recommended days of iron supplementation during pregnancy.

### Strengths and limitations

A nationally representative large data set and a rigorous statistical analysis method were used in this study. The sources of these secondary data were trustworthy. The study investigated the changes in adherence levels and the factors that contributed to them between the EDHSs 2005 and 2016. Despite its strength, this study did not include some important variables such as women's awareness of maternal anemia, perceptions/beliefs about not taking iron tablets, and side effects of iron supplement use by pregnant women. The cross-sectional design of the survey precludes the determination of cause-and-effect relationships.

## Conclusion

According to the EDHS, behavioral effects were responsible for nearly two-thirds (69.1%) of the overall change in the level of adherence, while compositional effects were responsible for one-third (30.9%) of the significant increase in the proportion of pregnant women who used iron supplements between 2005 and 2016. Most importantly, during the past 10 years, there has been a surge in the behavioral impacts of adherence due to women from wealthier families, older women, women with spouses with secondary or higher education, and women with a desire for conception.

Increases in the percentage of women who had four or more antenatal visits and increases in the number of women who were informed about pregnancy problems were compositional effects that helped improve the trend in the degree of adherence to taking iron supplements during pregnancy.

## Data availability statement

The original contribution presented in the study are included in the article/supplementary material, further inquiries can be directed to the corresponding author.

## Ethics statement

The studies involving human participants were reviewed and approved by University of Gondar's Institutional Review Boards. Written informed consent for participation was not required for this study in accordance with the national legislation and the institutional requirements.

## Author contributions

All authors contributed to the conceptualization, design, inception, data cleaning, data analysis, drafting of the first manuscript, manuscript approval, and read and approved the final version of the manuscript.
